# Cortisol, Perceived Stress, and Schema Modes in Adolescents: A Biopsychosocial Perspective

**DOI:** 10.1002/dev.70216

**Published:** 2026-11-06

**Authors:** Tatiane Trivilin, Rosa Maria Martins de Almeida

**Affiliations:** ^1^ Programa de Pós‐Graduação em Psicologia e Saúde Universidade Federal do Rio Grande do Sul (UFRGS) Porto Alegre Brazil; ^2^ Programa de Pós‐Graduação em Psicologia Universidade Federal do Rio Grande do Sul (UFRGS) Porto Alegre Rio Grande do Sul Brazil

**Keywords:** adolescent, cortisol, psychological, stress, Schema mode

## Abstract

Schema modes represent adaptive or maladaptive responses to life situations, integrating emotional, cognitive, and behavioral dimensions. Adolescence is a critical developmental stage marked by increased stress due to biological, psychological, and social changes. This study aimed to examine whether perceived stress and salivary cortisol are associated with the activation of schema modes in adolescents. A total of 73 participants aged 15–19 were divided into two groups: Group 0 (*n* = 37), without symptoms of perceived stress, and Group 1 (*n* = 36), with perceived stress symptoms. The findings confirmed that adolescents with higher perceived stress exhibited significantly greater activation of maladaptive schema modes. Additionally, elevated cortisol levels were associated with specific maladaptive modes. In contrast, healthy schema modes, particularly the happy child mode, were linked to lower levels of both perceived stress and cortisol. The perception of stress symptoms emerged as a key predictor for most of the schema modes that showed statistical significance, highlighting the importance of subjective stress appraisal in adolescent psychological functioning. These results underscore the relevance of stress perception in the activation of schema modes and suggest that strengthening healthy modes may be beneficial for promoting mental well‐being in adolescents.

## 1 Introduction

Schema modes are expressed as either adaptive or maladaptive responses to life situations, encompassing emotional, cognitive, and behavioral dimensions. In the context of addiction, these modes represent the predominant emotional states, underlying schemas, and coping mechanisms that become activated in an individual at a given moment (Grażka and Strzelecki 2023; Lobbestael and Arntz 2010; Young et al. 2006). Early maladaptive schemas (EMS) endure patterns of cognition, emotion, and memory that develop in response to adverse interactions with caregivers or traumatic experiences, ultimately frustrating core childhood needs. In contrast, schema modes refer to the currently activated clusters of schemas and associated coping strategies that emerge when EMS are triggered (Grażka and Strzelecki 2023; Stavropoulos et al. 2020).

Schema modes are organized into four macro categories that encompass both adaptive and maladaptive responses. The first category, child modes, reflects childhood experiences. Dysfunctional modes in this category include the vulnerable child, angry child, enraged child, impulsive child, and undisciplined child. The second category, parent modes, represents internalized parental criticism or demanding voices from attachment figures. Dysfunctional modes here include the punitive parent and the demanding parent (Lobbestael and Arntz 2010; Young et al. 2006). The third category, coping modes, includes strategies used to manage the internal conflict between child and parent modes. This category comprises five maladaptive coping modes: compliant surrender, detached protector, detached self‐soother, self‐aggrandizement, and bully and attack. (Lobbestael and Arntz 2010). Finally, healthy modes are associated with feelings of security, happiness, and love, and include the happy child and healthy adult modes(Lobbestael and Arntz 2010; Young et al. 2006).

Adolescence is a universally stressful period characterized by numerous decisions related to academic, social, and professional domains (Busler et al. 2022; Lindholdt et al. 2022). Additionally, during this developmental stage, individuals experience significant physiological changes such as puberty, fluctuations in self‐esteem (Wright et al. 2023), and brain development, particularly in the prefrontal cortex (Drzewiecki and Juraska 2020). Adolescence also marks the onset of the transition into adulthood (Wright et al. 2023) and the stress is conceptualized as the dynamic interaction between the individual and their environment (Corell et al. 2022; Wu et al. 2021) and adolescents, especially those aged between 15 and 19 years, are at increased risk for elevated stress levels (Wright et al. 2023).

There are two types of stress: biological and perceived stress. The biological stress response has been extensively studied with cortisol as the central hormone. Cortisol, the primary glucocorticoid produced in response to stress, plays a critical role in regulating neuronal survival and neurogenesis (Zajkowska et al. 2022). The hypothalamic–pituitary–adrenal (HPA) axis is activated during acute stress, resulting in the release of cortisol. While this activation is adaptive in the short term, chronic or prolonged stress—such as that caused by childhood maltreatment—can lead to dysregulation of the HPA axis, resulting in either heightened or blunted cortisol reactivity (Peckins et al. 2024). The second type of stress, perceived stress, is based on the individual's appraisal of a situation, rather than the objective nature of the event itself. This subjective interpretation explains variability in stress responses (Wu et al. 2021).

Although stress and schema modes have largely been investigated as separate constructs, they are conceptually connected. Schema modes are state‐like reactions that become activated when an individual is confronted with stressful or threatening situations; the appraisal of stress therefore functions as a proximal trigger that activates EMS and their associated coping responses (Grażka and Strzelecki 2023; Stavropoulos et al. 2020). Stress may operate through two complementary pathways: a biological pathway, indexed by HPA axis activity and cortisol output (Peckins et al. 2024), and a subjective pathway, reflected in the individual's perception of stress symptoms (Wu et al. 2021). Examining how these two pathways relate to schema‐mode activation is particularly relevant during adolescence, a developmental window in which the prefrontal cortex and stress‐response systems are still maturing (Drzewiecki and Juraska 2020), perceived stress is especially salient (Wu et al. 2021), and recurrently activated maladaptive modes may consolidate into more stable patterns that persist into adulthood. Clarifying these associations at this stage constituted the central rationale for the present research questions.

The activation of maladaptive schema modes is not a neutral phenomenon. These modes have been consistently associated with negative emotional states and psychological suffering, including heightened negative affect and emotional instability (Csukly et al. 2023), loneliness and depressive symptomatology (Reinhard et al. 2022), the differential expression of personality pathology (Mertens et al. 2020), and, in more severe presentations, histories of suicidality (Grażka and Strzelecki 2023). In adolescents, whose coping repertoires are still developing, the early entrenchment of such modes may represent a vulnerability factor for later mental health problems. Conversely, schema‐focused approaches aim to weaken maladaptive modes while strengthening healthy modes such as the healthy adult and happy child (Young et al. 2006), and evidence in adolescents and young people indicates that more adaptive coping strategies are linked to lower perceived stress and better psychosocial adjustment (Teixeira et al. 2022). Situating the present study within this framework highlights both the clinical relevance of examining stress‐mode associations and their potential to inform preventive and therapeutic interventions for adolescent mental health.

The objective of the present study was to examine whether perceived stress and salivary cortisol levels are associated with the activation of schema modes in adolescents. It was hypothesized that the perception of stress symptoms, encompassing cognitive, psychological, and physiological domains, would significantly predict the activation of maladaptive schema modes (Hypothesis 1). Additionally, it was anticipated that higher salivary cortisol levels would serve as predictors of schema mode activation (Hypothesis 2). This investigation aimed to contribute to a deeper understanding of the biopsychosocial mechanisms underlying adolescent stress responses and their relationship with EMS, potentially informing targeted mental health interventions for this population.

## 2 Methods

### 2.1 Participants

The participants in this study were part of a larger research project and were selected based on their level of perceived stress. Therefore, the sampling technique used was convenience sampling with specific inclusion criteria. A total of 73 adolescents participated in the study, with 37 in Group 0 (participants without symptoms of perceived stress) and 36 in Group 1 (participants with symptoms of perceived stress).

Group 0 included 16 males and 21 females aged between 15 and 18 years (*M* = 16.21), with 18 students in their first year of high school, 18 in the second year, and one in the third year. Group 1 included 14 males and 22 females aged between 15 and 19 years (*M* = 16.25), comprising 24 students in the first year and 12 in the second year of high school. (Table 1).

**TABLE 1:** Description of participants.

Variable	Group 0 (no perceived stress)	Group 1 (perceived stress)	Total
Total Participants (*N*)	37	36	73
Sex			
Male	16	14	30
Female	21	22	43
Age range (years)	15–18	15–19	15–19
Mean age (years)	16.21	16.25	—
School year			
First year	18	24	42
Second year	18	12	30
Third year	1	0	1

### 2.2 Tasks and Procedures

Data collection was conducted in three phases at the participants’ schools. In the first phase, an informational session was held to explain the study's objectives and procedures. During this session, informed consent forms were distributed to the students' legal guardians, along with assent forms for the adolescents themselves. After obtaining consent from both guardians and adolescents, tasks related to schema modes and perceived stress were administered during the second phase of data collection. Following this, participants were divided into two groups: Group 0 (without stress symptoms) and Group 1 (with stress symptoms). At the final stage of the study, both groups underwent salivary cortisol collection. The instruments used to collect data were as follows:

Young Schema Mode Inventory (SMI 1.1): This version includes 124 statements and assesses 16 schema modes. The instrument was translated and validated for the Brazilian population in 2020, with a Cronbach's alpha of 0.89 for the MED scale (Damasceno 2020).

Adolescent Stress Scale: This is a 5‐point Likert‐type scale that assesses the experience of stress responses as described in the statements. It consists of 44 items aimed at identifying the frequency and intensity of stress symptoms. The instrument provides two complementary classifications of stress responses. First, symptoms are organized into four domains: psychological, cognitive, physiological, and interpersonal. Second, stress responses are characterized according to four phases: alert, resistance, near‐exhaustion, and exhaustion. In the present study, analyses included the four symptom domains and the four stress phases, as well as the global symptom and phase indicators derived from the scale. The internal consistency, measured by Cronbach's alpha coefficient, was found to be satisfactory (*α* = 0.89) (Tricoli and Lipp 2005).

Elabscience Human Cortisol ELISA Kit: This ELISA kit is used for the in vitro quantitative determination of human cortisol concentrations in serum, plasma, and other biological fluids.

Group classification was based on participants’ scores on the Adolescent Stress Scale. Adolescents whose overall stress symptomatology fell within the expected range for their age were allocated to Group 0 (no perceived stress symptoms), whereas those whose scores exceeded the scale's reference threshold for clinically relevant stress were allocated to Group 1 (perceived stress symptoms), following the normative cut‐off criteria established for the instrument (Tricoli and Lipp 2005). Perceived stress was chosen as the grouping variable, rather than cortisol or a combination of both indicators, because the central aim of the study was to test whether the subjective appraisal of stress—as opposed to a single biological measurement—predicts schema‐mode activation; in addition, salivary cortisol reflects a much narrower temporal window and shows considerable inter‐individual variability during adolescence, which makes it less suitable as a stable classification criterion.

Data were collected across three sequential phases for logistical and methodological reasons. The self‐report instruments (schema modes and perceived stress) were administered during the second phase, and only after participants had been classified into groups were salivary samples obtained in the third phase, so that cortisol collection could be organized around the resulting groups. The interval between the second and third phases was kept as short as the school schedule allowed, within the same data‐collection period, in order to minimize changes in participants’ circumstances between the two assessments.

Salivary samples were collected at a single time point during the third phase. Collection took place in the morning, after participants had arrived at school, within a standardized time window that was kept consistent across all participants in order to limit circadian variability in cortisol. Samples were obtained at school rather than immediately upon awakening because the study was conducted entirely within the school setting, which precluded a home‐based awakening protocol. For the same reason, a single‐sample design was adopted instead of a multipoint protocol (e.g., repeated questionnaire administrations or cortisol sampling from awakening up to 60 min post‐awakening), although a multipoint design would better capture diurnal dynamics and the cortisol awakening response, it was not feasible within the school‐based, single‐visit logistics of the present project. Cortisol concentrations were subsequently quantified from the saliva samples using the Elabscience Human Cortisol ELISA Kit according to the manufacturer's instructions, and the resulting concentration values were used in the statistical analyses.

It should also be noted that the two stress indicators capture different temporal windows. Salivary cortisol reflects the biological stress response over the minutes to hours preceding collection, whereas the Adolescent Stress Scale assesses the frequency and intensity of stress symptoms experienced more generally over the preceding period (Tricoli and Lipp 2005). The two measures are therefore complementary rather than directly interchangeable, and this distinction is taken into account when interpreting their respective associations with schema‐mode activation.

### 2.3 Statistical Analysis

Statistical analyses were performed using the Mann–Whitney *U* test, which revealed statistically significant differences between groups with and without perceived stress across several schema modes and stress indicators. Additionally, linear regression analyses were conducted. Model 1 included cortisol levels, group membership, sex, and global stress indicators as predictors of schema mode activation. Model 2 included specific stress dimensions as predictors.

For clarity, two prediction models were specified for each schema mode. Model 1 included cortisol level, group membership, sex, and the global stress indicators as predictors, whereas Model 2 included the specific stress dimensions (the psychological, cognitive, physiological, and interpersonal symptom domains and the alert, resistance, near‐exhaustion, and exhaustion phases). The standardized coefficients and fit indices for both prediction models are reported in Table 4. Given the large number of variables examined in this study, Table 2 summarizes and categorizes them by domain, subcategory, and instrument, to orient the reader before the descriptive and inferential results are presented.

**TABLE 2:** Overview and categorization of study variables.

Domain	Subcategory	Variables
Schema modes (Young Schema Mode Inventory, SMI 1.1)	Child modes	Vulnerable child, angry child, enraged child, impulsive child, undisciplined child
	Parent modes	Punitive parent, demanding/critical parent
	Coping modes	Compliant surrender, detached protector, detached self‐soother, self‐aggrandizer, bully and attack
	Healthy modes	Happy child, healthy adult
Perceived stress (Adolescent Stress Scale)	Global indicators	Stress determination—symptom; stress indicator—phase
	Symptom domains	Psychological, cognitive, physiological, interpersonal
	Stress phases	Alert, resistance, near‐exhaustion, exhaustion
Biological stress marker	—	Salivary cortisol (Elabscience Human Cortisol ELISA Kit)

*Note:* Schema modes were assessed with the Young Schema Mode Inventory (SMI 1.1; Damasceno 2020); perceived stress with the Adolescent Stress Scale (Tricoli and Lipp 2005); and salivary cortisol with the Elabscience Human Cortisol ELISA Kit. Global Indicators refer to the overall symptom and phase scores derived from the Adolescent Stress Scale.

## 3 Results

### 3.1 Group Differences in Perceived Stress, Cortisol, and Schema Modes

Participants with activated stress perception (Group 1) exhibited significantly higher scores in maladaptive schema modes, including vulnerable child (*U* = 195.5, *p* < 0.001), enraged child (*U* = 402.5, *p* = 0.004), angry child (*U* = 412.0, *p* = 0.005), impulsive child (*U* = 368.0, *p* = 0.001), and undisciplined child (*U* = 408.5, *p* = 0.004). They also showed elevated scores in the coping modes of compliant surrender (*U* = 393.0, *p* = 0.003), detached protector (*U* = 228.5, *p* < 0.001), and self‐aggrandizement (*U* = 438.5, *p* = 0.012), as well as in the punitive parent mode (*U* = 365.0, *p* < 0.001).

The healthy adult mode presented significantly lower scores in the group with activated stress perception (*U* = 375.5, *p* = 0.001). Moreover, highly significant differences were observed in stress indicators. Participants with activated stress perception reported higher levels of psychological, cognitive, physiological, and interpersonal symptoms (all *p* < 0.001), as well as a greater prevalence of the stress phases of alert, resistance, near‐exhaustion, and exhaustion (all *p* < 0.001).

### 3.2 Descriptive Statistics of Schema Modes and Stress Subscales

Descriptive statistics (mean, median, standard deviation, and standard error) for each schema mode, for salivary cortisol, and for each of the four subscales of the Adolescent Stress Scale (psychological, cognitive, physiological, and interpersonal symptoms), as well as for the global symptom and phase indicators, are presented separately for each group in Table 3.

**TABLE 3:** Descriptive statistics of the groups.

	Group	*N*	Mean	Median	Standard deviation	Standard error
Vulnerable child	0	37	2.02	1.90	0.833	0.1370
	1	36	3.80	4.20	1.323	0.221
Angry child	0	37	2.99	2.50	1.250	0.2054
	1	36	3.82	3.80	1.145	0.191
Enraged child	0	37	1.99	1.60	1.239	0.2036
	1	36	2.51	2.35	1.015	0.169
Impulsive child	0	37	2.59	2.11	1.179	0.1939
	1	36	3.47	3.17	1.165	0.194
Undisciplined child	0	37	3.09	3.00	0.942	0.1548
	1	36	3.74	3.83	1.116	0.186
Happy child	0	37	4.08	4.20	0.795	0.1307
	1	36	2.91	2.90	0.857	0.143
Compliant surrender	0	37	2.83	2.71	0.864	0.1420
	1	36	3.52	3.64	1.077	0.180
Detached protector	0	37	1.97	1.89	0.821	0.1350
	1	36	3.24	3.28	1.092	0.182
Detached self‐soother	0	37	3.61	3.75	1.432	0.2354
	1	36	4.44	4.63	1.037	0.173
Self‐aggrandizement	0	37	3.08	3.10	1.207	0.1984
	1	36	3.17	3.20	1.085	0.181
Bully and attack	0	37	2.62	2.33	1.023	0.1682
	1	36	2.76	2.39	0.923	0.154
Punitive parent	0	37	1.96	1.90	0.778	0.1279
	1	36	2.68	2.55	0.919	0.153
Demanding/critical parent	0	37	3.99	3.90	0.958	0.1575
	1	36	4.14	4.00	0.864	0.144
Healthy adult	0	37	4.57	4.70	0.602	0.0989
	1	36	4.04	4.15	0.750	0.125
Cortisol	0	37	7.53	3.89	9.478	1.5582
	1	36	5.11	3.76	5.538	0.923
Stress determination—symptom	0	37	2.17	2.20	0.489	0.0805
	1	36	3.41	3.45	0.675	0.112
Stress indicator—phase	0	37	2.12	2.09	0.486	0.0799
	1	36	3.41	3.51	0.648	0.108
Predominant symptom—psychological	0	37	1.89	1.92	0.973	0.1599
	1	36	3.04	3.44	1.412	0.235
Predominant symptom—cognitive	0	37	2.15	2.17	0.700	0.1151
	1	36	3.44	3.58	0.901	0.150
Predominant symptom—physiological	0	37	2.08	2.11	0.560	0.0920
	1	36	3.15	3.00	0.786	0.131
Predominant symptom—interpersonal	0	37	2.09	2.00	0.698	0.1148
	1	36	3.28	3.40	1.029	0.172
Predominant phase—alert	0	37	2.17	2.05	0.647	0.1063
	1	36	3.49	3.50	0.773	0.129
Predominant phase—resistance	0	37	2.04	1.89	0.593	0.0974
	1	36	3.49	3.56	0.800	0.133
Predominant phase—near‐exhaustion	0	37	2.09	2.00	0.592	0.0973
	1	36	3.13	3.09	0.796	0.133
Predominant phase—exhaustion	0	37	2.14	2.20	0.787	0.1294
	1	36	3.42	3.20	0.903	0.150

Table 3 presents the descriptive statistics of the groups along with the corresponding statistical differences.

### 3.3 Prediction of Schema Mode Activation: Linear Regression Models

The regression findings below follow the order established in the preceding paragraphs; within each mode, results are identified according to which hypothesis they address. In brief, the perception of stress symptoms (Hypothesis 1) emerged as a significant predictor across most modes with an adequate model fit, vulnerable child, enraged child, impulsive child, detached protector, and punitive parent (negatively for happy child), whereas salivary cortisol (Hypothesis 2) was a significant predictor for a narrower subset, namely the vulnerable child and enraged child modes.

Regarding child modes, the vulnerable child mode showed a good fit in Model 1 (adjusted *R* ^2^ = 0.675, *F*(5,67) = 30.9, *p* < 0.001). In this model, lower cortisol levels (*β* = −0.0361, *p* = 0.005) and higher perception of stress symptoms (*β* = 1.3992, *p* < 0.001) were significant predictors of increased activation of this schema mode (cortisol supporting H2 and stress‐symptom perception supporting H1). Model 2 explained 68.5% of the variance in the dependent variable (adjusted *R* ^2^ = 0.685, *F*(10,62) = 16.6, *p* < 0.001), with physiological symptoms (*β* = 0.5402, *p* = 0.037), the resistance phase (*β* = 0.7237, *p* = 0.003), and the near‐exhaustion phase, with a negative association (*β* = −0.5484, *p* = 0.043), emerging as significant predictors consistent with H1.

For the enraged child mode, Model 1 also showed a good fit (adjusted *R* ^2^ = 0.456, *F*(5,67) = 13.1, *p* < 0.001), and Model 2 explained 36.2% of the variance in the dependent variable (adjusted *R* ^2^ = 0.362, *F*(10,62) = 5.09, *p* < 0.001). Higher cortisol levels (*β* = 0.0324, *p* = 0.027) and greater perception of stress symptoms (*β* = 1.3388, *p* < 0.001) were significant predictors of the activation of this mode (cortisol supporting H2 and stress‐symptom perception supporting H1), while being in Group 1 was a significant negative predictor of activation (*β* = −1.0449, *p* = 0.012) in Model 1. In Model 2, cognitive symptoms (*β* = 0.7993, *p* = 0.003), the alert phase (*β* = 0.9219, *p* < 0.001), and, inversely, the exhaustion phase (*β* = −0.5711, *p* = 0.035) were significant predictors.

The impulsive child mode showed a moderate fit in Model 1 (adjusted *R* ^2^ = 0.314, *F*(5,67) = 7.60, *p* < 0.001), with perception of stress symptoms as the only significant predictor (*β* = 0.7435, *p* = 0.014), consistent with H1. Model 2 demonstrated a good fit (adjusted *R* ^2^ = 0.352, *F*(10,62) = 4.91, *p* < 0.001), with the alert phase being the only significant predictor (*β* = 1.0359, *p* < 0.001). The undisciplined child and angry child modes showed a poor, nonsignificant model fit in either Model 1 or Model 2.

Among the dysfunctional coping modes, the detached protector mode showed a significant fit in Model 1 (adjusted *R* ^2^ = 0.455, *F*(5,67) = 13.0, *p* < 0.001). Perception of stress symptoms was a significant predictor of this mode (*β* = 1.132, *p* < 0.001), consistent with H1. When specific stress determinants were included in Model 2, the fit improved considerably (adjusted *R* ^2^ = 0.563, *F*(10,62) = 10.3, *p* < 0.001). Physiological symptoms (*β* = 0.572, *p* = 0.022) and the predominant resistance phase (*β* = 0.467, *p* = 0.040) were significant predictors. Linear regression analyses for the self‐aggrandizement, compliant surrender, detached self‐soother, and bully and attack modes showed a poor, nonsignificant model fit in both models.

Regarding dysfunctional parent modes, the punitive parent mode demonstrated statistical significance in Model 1, which included cortisol, group, sex, and global stress indicators (adjusted *R* ^2^ = 0.266, *F*(5,67) = 6.21, *p* < 0.001). The perception of stress symptoms was a significant predictor (*β* = 0.524, *p* = 0.023), consistent with H1. When specific stress determinants were added in Model 2, the model's fit improved while remaining statistically significant (adjusted *R* ^2^ = 0.309, *F*(10,62) = 4.22, *p* < 0.001). The only statistically significant predictor in Model 2 was the predominant resistance phase (*β* = 0.514, *p* = 0.025). The linear regression analysis for the demanding/critical parent mode showed a poor, nonsignificant model fit in both models.

Finally, among the healthy modes, the happy child mode showed a good fit in Model 1 (adjusted *R* ^2^ = 0.461, *F*(5,67) = 13.3, *p* < 0.001) and also in Model 2 (adjusted *R* ^2^ = 0.451, *F*(10,62) = 6.92, *p* < 0.001). The perception of stress symptoms was a significant negative predictor of this mode (*β* = −0.9249, *p* < 0.001), consistent with H1. The predominant phase indicator was also a significant positive predictor (*β* = 0.4235, *p* = 0.046). In Model 2, Group 1 and physiological symptoms were significant negative predictors (*β* = −0.6688, *p* = 0.045; *β* = −0.5057, *p* = 0.038, respectively). The healthy adult mode showed a poor, nonsignificant model fit (Table 4).

**TABLE 4:** Linear regression results.

Schema mode	Model	*R* ^2^ aju.	*F*(*d*)	*p*	*β* (predictor)	*p*
Vulnerable child	Model 1	0.675	*F*(5, 67) = 30.9	< 0.001	Cortisol (−0.0361)	0.005
					Stress symptoms (1.3992)	< 0.001
	Model 2	0.685	*F*(10, 62) = 16.6	< 0.001	Physiological symptoms (0.5402)	0.037
					Resistance phase (0.7237)	0.003
					Near‐exhaustion phase (−0.5484)	0.043
Enraged child	Model 1	0.456	*F*(5, 67) = 13.1	< 0.001	Cortisol (0.0324)	0.027
					Stress symptoms (1.3388)	< 0.001
					Group (−1.0449)	0.012
	Model 2	0.362	*F*(10, 62) = 5.09	< 0.001	Cognitive symptoms (0.7993)	0.003
					Alert phase (0.9219)	< 0.001
					Exhaustion phase (−0.5711)	0.035
Impulsive child	Model 1	0.314	*F*(5, 67) = 7.60	< 0.001	Stress symptoms (0.7435)	0.014
	Model 2	0.352	*F*(10, 62) = 4.91	< 0.001	Alert phase (1.0359)	< 0.001
Detached protector	Model 1	0.455	*F*(5, 67) = 13.0	< 0.001	Stress symptoms (1.132)	< 0.001
	Model 2	0.563	*F*(10, 62) = 10.3	< 0.001	Physiological symptoms (0.572)	0.022
					Resistance phase (0.467)	0.040
Punitive parent	Model 1	0.266	*F*(5, 67) = 6.21	< 0.001	Stress symptoms (0.524)	0.023
	Model 2	0.309	*F*(10, 62) = 4.22	< 0.001	Resistance phase (0.514)	0.025
Happy child	Model 1	0.461	*F*(5, 67) = 13.3	< 0.001	Stress symptoms (−0.9249)	< 0.001
	Model 2	0.451	*F*(10, 62) = 6.92	< 0.001	Group (−0.6688)	0.045
					Physiological symptoms (−0.5057)	0.038
					Phase stress (0.4235)	0.046

## 4 Discussion

### 4.1 Perceived Stress and the Activation of Maladaptive Schema Modes

In the present study, participants with elevated levels of perceived stress exhibited significantly higher activation of schema modes compared to those without above‐average stress perception for their age group. Similarly, in a study by Stavropoulos et al. (2020), participants who reported higher levels of worry also demonstrated increased activation of schema modes, whereas those with lower levels of worry exhibited fewer activated schemas.

Data analysis revealed that lower cortisol levels significantly predicted activation of the maladaptive vulnerable child schema mode. Reduced cortisol has been associated with depressive and anxious symptomatology, as individuals exposed to prolonged stress may experience long‐term cortisol attenuation. While lower biological stress was negatively associated with vulnerable child activation, increased perceived stress was positively associated with this mode. Furthermore, physiological stress symptoms, like physical responses to stress such as headaches, sleep disturbances, fatigue, appetite changes, and muscle tension experienced in the past week (Mendes et al. 2011), were found to be important factors contributing to the activation of the vulnerable child mode. However, when stress reached an intermediate stage between resistance and exhaustion, a phase characterized by a decline in the organism's capacity to cope with stressors, activation of the vulnerable child mode, which typically engages the sensitive and wounded aspect of the self‐associated with feelings of sadness, fear, and loneliness (Lobbestael and Arntz 2010; Mertens et al. 2020), was not observed. Moreover, this schema mode is among the most frequently activated child modes in clinical samples, indicating a strong association between its activation and emotional suffering (Lobbestael and Arntz 2010).

The perception of stress symptoms, that is, the extent to which an individual somatically, psychologically, or socially recognizes stress in their daily life, was found to be a significant predictor of activation of several maladaptive schema modes, not only the vulnerable child but also the enraged child, impulsive child, detached protector, and punitive parent. There is a well‐established and direct relationship between maladaptive schema modes and negative emotions (Csukly et al. 2023). Furthermore, associations between maladaptive modes and worry tend to diminish when linked to experiential avoidance (Stavropoulos et al. 2020). This avoidance is particularly evident in the impulsive child mode, which seeks immediate pleasure or gratification without regard for rules, limits, or consequences, and in the detached protector mode, which is often regarded as a dysfunctional coping mechanism (Reinhard et al. 2022) The detached protector is characterized by emotional disconnection as a means of self‐protection and reflects a disengagement from social relationships to shield oneself from painful experiences, such as rejection (Lobbestael and Arntz 2010; Reinhard et al. 2022). In relation to this last schema mode, perceptions of vulnerability and suffering may activate dysfunctional coping modes as a defensive strategy to prevent the activation of maladaptive child modes (Grażka and Strzelecki 2023).

### 4.2 Specific Child, Coping, and Parent Modes

Regarding the maladaptive child modes, the enraged child mode, characterized by anger that escalates to explosive and uncontrolled proportions, resulting in aggressive and destructive behaviors (Lobbestael and Arntz 2010) was found to be more highly activated in association with elevated cortisol levels and the perception of cognitive stress symptoms. Cognitive stress involves alterations in mental processes such as difficulties with concentration, memory lapses, recurrent negative thoughts, and indecisiveness. This type of stress perception can adversely affect performance on cognitive tasks and decision‐making (Mendes et al. 2011). Interestingly, activation of the enraged child mode decreased when individuals reported high levels of exhaustion‐related stress symptoms over the past month (Mendes et al. 2011). This finding suggests that individuals may engage in avoidant schema modes or contradictory behaviors to diminish awareness of their emotional distress (Reinhard et al. 2022).

The impulsive child mode was primarily predicted by stress perception, as previously described, as well as by the alert phase, which encompasses physiological, physical, and psychological symptoms (Mendes et al. 2011). Finally, the predominantly resistant phase was a significant predictor of schema activation for both the punitive parent and detached protector modes, with physiological symptoms also serving as important predictors for the activation of the latter.

### 4.3 Healthy Modes as Protective Factors

Concerning the happy child schema mode, the individual experiences feelings of calmness, safety, care, and connectedness with others. With a sense of fulfillment and self‐worth, they are able to indulge in optimism, spontaneity, and contentment (Grażka and Strzelecki 2023). Notably, findings suggest that lower overall perception of stress symptomatology is associated with greater activation of this schema mode. Similarly, significant negative association observed for Group 1, indicating that participants with higher levels of stress symptomatology exhibited reduced activation of the happy child mode. In a study conducted with university students, Teixeira et al. (2022) found that students who employed more adaptive coping strategies perceived challenges more effectively, experienced less stress, and demonstrated better environmental adjustment.

### 4.4 The Role of Cortisol

It is important to note that cortisol did not show statistical significance when comparing differences between groups. However, in individual analyses of each schema mode, cortisol demonstrated an interesting association with both the vulnerable child and enraged child modes. Adolescence is a highly dynamic period characterized by a gradual distancing from parental influence and an increase in autonomy (Steen et al. 2020). This developmental stage also exhibits heterogeneity in cortisol responses to stress (Peckins et al. 2024), which may explain the lack of statistical significance in cortisol levels between groups as assessed by the Mann–Whitney test. Additionally, it is noteworthy that the subgroup with higher activation of the happy child mode exhibited lower perceived stress and significantly lower cortisol levels. Thus, strengthening the healthy schema mode may serve as a protective strategy to reduce activation of maladaptive schema modes and both biological and perceived stress (Teixeira et al. 2022).

## 5 Conclusions

The results of the study support that the perception of stress symptoms (H1) and, to a more limited extent, salivary cortisol levels (H2) were significant predictors of greater activation of maladaptive schema modes, indicating that adolescents who appraise their circumstances as more stressful, and who present certain patterns of cortisol, are more likely to show heightened activation of these modes. Additionally, healthy modes, such as the happy child mode, were associated with lower perceived stress and reduced cortisol levels.

The study presented certain limitations that should be acknowledged. First, it is important to note that data collection took place in the year 2024, in the city of Porto Alegre, located in the southern region of Brazil. This area experienced severe flooding during the winter season, and many residents lost their homes and were forced to live in shelters or with relatives. These circumstances may have influenced both perceived stress and cortisol levels. Another important consideration is that the salivary cortisol samples were collected only once and within the participants’ school environment, rather than immediately upon waking. Therefore, cortisol levels may have been affected during the commute to school.

Nevertheless, the perception of stress symptoms emerged as a significant predictor for nearly all schema modes that reached statistical significance in the sample, indicating its relevance as a factor that should be carefully observed in adolescents to promote better mental health. Strengthening protective modes in children and adolescents is also a means of fostering healthier developmental trajectories into adulthood. This topic, which is highly relevant to psychological development, remains underexplored in research and warrants greater attention so that new intervention strategies targeting stress can be developed in collaboration with professionals working in adolescent mental health care.

### 5.1 Implications for Adolescent Mental Health

The present findings carry direct implications for adolescent mental health practice. Because the subjective perception of stress, rather than cortisol alone, emerged as the most consistent predictor of maladaptive schema‐mode activation, screening for perceived stress in school and clinical settings may help identify adolescents at greater risk of mode‐related emotional difficulties. Interventions could prioritize teaching adaptive stress‐appraisal and coping skills, which are associated with lower perceived stress and better adjustment in young people (Teixeira et al. 2022), as well as schema‐focused strategies that deliberately strengthen healthy modes such as the happy child and healthy adult while reducing the activation of maladaptive modes (Young et al. 2006). Given that the happy child mode was linked here to lower perceived stress and lower cortisol, fostering positive experiences, supportive relationships, and emotion‐regulation skills may serve as a protective, preventive avenue for promoting adolescent well‐being.

## Author Contributions

*Conception or design*: Tatiane Trivilin and Rosa Maria Martins de Almeida. *Collection of data*: Tatiane Trivilin. *Analysis of data*: Tatiane Trivilin. *Interpretation of data*: Tatiane Trivilin and Rosa Maria Martins de Almeida. *Drafting the article*: Tatiane Trivilin. *Revising the article critically for important intellectual content*: Tatiane Trivilin and Rosa Maria Martins de Almeida.

## Funding

The authors have nothing to report.

## Ethics Statement

All procedures performed in studies involving human participants were in accordance with the ethical standards of the Brazilian National Health Council (approval no. CAEE: 69848423.2.0000.5334) and with the 1964 Helsinki Declaration and its later amendments or comparable ethical standards.

## Conflicts of Interest

The authors declare no conflicts of interest.

## Data Availability

The data that support the findings of this study are available from the corresponding author upon reasonable request.
